# Radical Treatment of Hernia by Excision of the Sac

**Published:** 1884-09

**Authors:** William McCormac


### Radical Treatment of Hernia by Excision of the Sac.

The radical cure of hernia is a subject of much practical interest. It consists of the partial or complete excision of the hernial sac and the subsequent suturing of the pillars of the inguinal canal, and the closure, after a similar fashion, of the cervical ring. This plan is really very old, although it has been recently introduced under new conditions, and thus may be still considered on its trial. Sir William McCormac reports a series of sixteen cases in which this plan of operation was used, with the following results: In thirteen cases there was complete cure and in one case a recurrence of the hernia. There were, besides, two fatal cases, but these were from causes unconnected with the operation. In one of the fatal cases the patient was a female, aged 50, who had suffered from femoral hernia of the right side for thirty years. She had symptoms of incomplete strangulation for five days preceding the operation, when, with antiseptic precautions, the hernia was reduced, and the sac and some surplus skin removed. She died six days afterwards from œdema of the lungs and slight incipient peritonitis. The other case was a man aged 69, who had had a left inguinal hernia for fifteen years, when, four days before the operation was made, in consequence of a sudden strain, a swelling appeared in the right groin, which proved to be a femoral hernia. Symptoms of strangulation followed immediately. The hernia was reduced and the sac ligatured with catgut at the neck and removed. Vomiting and hiccough supervened and continued until the death of the patient, two days afterwards, from peritonitis. A portion of the strangulated intestine was found to have sloughed. The bowel below was empty and collapsed.

Of the other cases, eleven were inguinal, including one double hernia, and three were femoral; twelve were males and two were females. The time when the permanence of the cure was examined into varied from a few weeks or months, in the two most recent, to four years and eight months in the oldest of the cases reported. In all of these cases, with one exception, the cure was complete, most of the patients going without a truss, and being able to attend to their usual vocations. In the case where the hernia returned, the operation had been successful and the patient discharged cured, but as he was leaving the hospital he had a severe fall and the rupture then and there reappeared, and has remained down ever since. In commenting upon this successful series of cases, the author concludes, first, that in all cases of strangulated hernia, the usual operation for the division of the stricture having been performed, partial or complete abcision of the sac should be likewise done as an ordinary part of the operation. Where the aperture is large, its margins should be drawn together by suture. Chromised or green catgut admirably suffices for the purpose, or, if preferred, silver sutures may be used. The use of antiseptics is strictly enjoined in every step of the operation. Second, a similar operation is justifiable in cases of hernia, either femoral or inguinal, which cause the patient pain and inconvenience, or which are not retained by a truss when the individual is about his ordinary vocations. If the patient be in good general health it may be performed without serious risk, and with the result, probably, of permanently freeing the individual from the consequences of a debilitating infirmity.—*Sir William McCormac, in British Medical Journal, August* 2, 1884.

